# Rapid In Vivo Validation of HDAC Inhibitor-Based Treatments in Neuroblastoma Zebrafish Xenografts

**DOI:** 10.3390/ph13110345

**Published:** 2020-10-27

**Authors:** Jagoda K Wrobel, Sara Najafi, Simay Ayhan, Charlotte Gatzweiler, Damir Krunic, Johannes Ridinger, Till Milde, Frank Westermann, Heike Peterziel, Benjamin Meder, Martin Distel, Olaf Witt, Ina Oehme

**Affiliations:** 1 Hopp Children’s Cancer Center Heidelberg (KiTZ), 69120 Heidelberg, Germany; jkl.wrobel@gmail.com (J.K.W.); s.najafi@kitz-heidelberg.de (S.N.); s.ayhan@kitz-heidelberg.de (S.A.); charlotte.gatzweiler@dkfz-heidelberg.de (C.G.); j.ridinger@kitz-heidelberg.de (J.R.); t.milde@kitz-heidelberg.de (T.M.); f.westermann@kitz-heidelberg.de (F.W.); h.peterziel@kitz-heidelberg.de (H.P.); o.witt@kitz-heidelberg.de (O.W.); 2 Clinical Cooperation Unit Pediatric Oncology, German Cancer Consortium (DKTK), German Cancer Research Center (DKFZ), 69120 Heidelberg, Germany; 3 Faculty of Medicine, Heidelberg University, 69120 Heidelberg, Germany; 4 Faculty of Biosciences, Heidelberg University, 69120 Heidelberg, Germany; 5 Light Microscopy Facility, German Cancer Research Center (DKFZ), 69120 Heidelberg, Germany; D.Krunic@dkfz-heidelberg.de; 6 Department of Pediatric Oncology, Hematology and Immunology, University of Heidelberg Medical Center, 69120 Heidelberg, Germany; 7 Neuroblastoma Genomics, German Cancer Research Center (DKFZ), 69120 Heidelberg, Germany; 8 Institute for Cardiomyopathies Heidelberg, Heidelberg University, 69120 Heidelberg, Germany; Benjamin.Meder@med.uni-heidelberg.de; 9 Genome Technology Center, Stanford University, Stanford, CA 94304, USA; 10 Innovative Cancer Models, St. Anna Children’s Cancer Research Institute, 1090 Vienna, Austria; martin.distel@ccri.at

**Keywords:** childhood cancer, preclinical models, precision medicine, xenotransplantation, small molecules, histone deacetylases

## Abstract

The survival rate among children with relapsed neuroblastomas continues to be poor, and thus new therapeutic approaches identified by reliable preclinical drug testing models are urgently needed. Zebrafish are a powerful vertebrate model in preclinical cancer research. Here, we describe a zebrafish neuroblastoma yolk sac model to evaluate efficacy and toxicity of histone deacetylase (HDAC) inhibitor treatments. Larvae were engrafted with fluorescently labeled, genetically diverse, established cell lines and short-term cultures of patient-derived primary cells. Engrafted tumors progressed locally and disseminated remotely in an intact environment. Combination treatments involving the standard chemotherapy doxorubicin and HDAC inhibitors substantially reduced tumor volume, induced tumor cell death, and inhibited tumor cell dissemination to the tail region. Hence, this model allows for fast, cost-efficient, and reliable in vivo evaluation of toxicity and response of the primary and metastatic tumor sites to drug combinations.

## 1. Introduction

In recent years, studies exploiting zebrafish (*Danio rerio*) as a disease model led to the identification of several promising therapeutic targets and drug candidates currently being tested in preclinical or late-phase clinical trials [1,2,3,4,5,6,7]. Engraftment of zebrafish with human tumor cells is rapidly gaining recognition as a valid technique that is suitable for preclinical drug screening in a cost- and time-effective manner [8,9,10,11,12]. Transparency and the lack of a fully functional adaptive immune system in the first weeks of life are two characteristics that make zebrafish larvae highly valuable for xenotransplantation approaches [13,14,15]. Specific pigmentation inhibitors such as 1-phenyl-2-thiourea (PTU) or the usage of pigmentation mutants can prolong optical transparency. Zebrafish xenograft models require implantation of no more than a few hundred cells per fish [16,17], thus making them a particularly attractive model for studies involving patient-derived material [9]. The most common sites of injection are the yolk sac [9,14], which provides a nutrient rich environment and can accommodate a relatively high volume of injected material [11,13]; the avascular perivitelline space or pericardium, which are preferred for metastasis and angiogenesis studies; and the brain, with such injections being employed to study the behavior of tumor cells in the brain microenvironment [17,18,19,20,21,22]. 

Childhood tumors of the central and peripheral nervous system account for almost one-third of all total cancer cases diagnosed in children. Neuroblastoma is the most common, yet heterogeneous, extracranial pediatric solid tumor deriving from the developing sympathoadrenal lineage of neural crest cells [23]. It is characterized by an extremely low survival rate following metastatic relapse [23,24,25]. Although major efforts over the past decades have resulted in great advancement in our understanding of the genetic and molecular background of the disease, the outcomes among high-risk neuroblastoma patients continue to be poor, with 5-year overall survival below 50% and a median progression-free survival of 6–7 months [26,27,28]. Low therapeutic success rates, largely attributed to neuroblastoma heterogeneity and associated chemotherapy resistance, emphasize the urgent need for strategies that accelerate the selection of the most promising drug candidates and treatment combinations to improve therapy precision [23,24,26,29]. 

Histone deacetylases (HDACs) are an enzyme family involved in a number of fundamental cellular processes and play a pivotal role in childhood cancers of the nervous system [30,31,32,33,34]. We and others have previously demonstrated that abnormal HDAC expression or activity is linked to oncogenic events. Furthermore, we and others have proven the association between selective HDAC inhibition and various antitumor effects, e.g., cell death or cell cycle arrest [29,35,36,37,38]. To date, several HDAC inhibitors (HDACis) have been integrated into treatment for hematological cancers (e.g., vorinostat for cutaneous T-cell lymphoma, panobinostat for multiple myeloma), and clinical trials investigating their utility in solid tumors, both as single agents and in combination with chemotherapeutics, are ongoing [30,39,40].

The present study firstly established zebrafish xenograft models of neuroblastoma tumors, which provides an intact environment to study disease progression, including metastatic dissemination, more closely resembling human pediatric tumor patients. Secondly, it was demonstrated that specific therapeutic interventions can be evaluated using this in vivo model. The results confirm that our zebrafish xenograft model serves as a feasible tool to investigate new combination therapies and obtain fast, reliable results on drug efficacy and toxicity, which may reduce the need for subsequent mammalian studies. 

## 2. Results

### 2.1. Neuroblastoma Cells Proliferate within Yolk Sac Zebrafish Xenografts 

Short-term cultures of primary neuroblastoma patient samples (HD-N33, NB-S-124) and cells of one established cell line (SK-N-BE(2)-C), were engrafted into the yolk sac of zebrafish embryos (Table 1, Figure S1a). 

**Figure 1:**
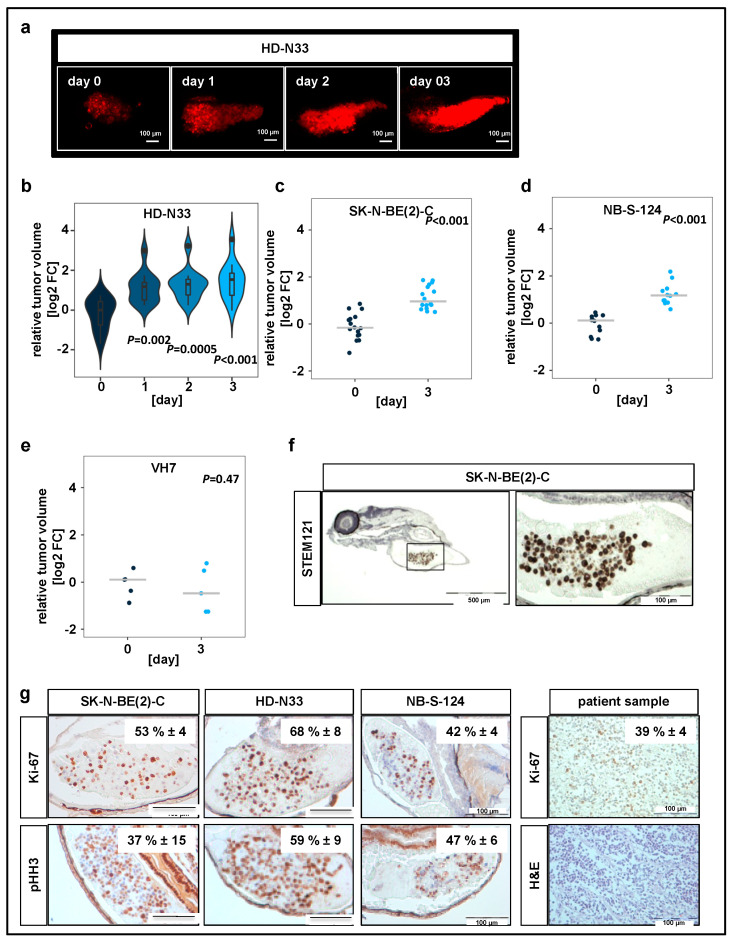
Neuroblastoma cell lines proliferate in zebrafish xenografts. (**a**) Zebrafish larvae were imaged using confocal microscopy on the day when injections were performed (day 0), as well as on days 1, 2, and 3 after tumor cell implantation. Representative tile region images of larvae injected with a suspension of fluorescently labeled HD-N33 cells, tumor cells appear red. Scale bar = 100 µm. (**c**) Tumor progression was monitored by automated quantification of tumor volume. Violin plots for HD-N33 cells, *n* = 12 larvae, ANOVA with Tukey’s multiple comparison test. (**b**–**e**) Strip charts for SK-N-BE(2)-C ((**c**), *n* = 16 larvae), NB-S-124 ((**d,**) *n* = 12 larvae) and nonmalignant VH7 ((**e**), *n* = 5 larvae) cells, one sample t-test. (**f**) Zebrafish larvae engrafted with SK-N-BE(2)-C cells (48 h post implantation (hpi)) were stained with α-STEM121, confirming cells of human origin. Scale bar = 500 µm (left) and 100 µm (right). (**g**) Representative images of immunohistochemistry (IHC) staining for Ki-67 and phospho-histone H3 in zebrafish engrafted with SK-N-BE(2)-C, HD-N33, or NB-S-124 cells (48 hpi), as well as Ki-67 staining and H&E staining of slides obtained from neuroblastoma patients. Inlay: quantified results. *n* = 3–7 fish per group and *n* = 4 neuroblastoma patients, values are mean positive cells/slide in % ± SD, scale bar = 100 μm.

Nonmalignant cells, namely VH7 fibroblasts, were used to verify whether our observations on tumor progression in zebrafish were specific to tumor cells. All yolk-sac-injected tumor cell lines, but not the nonmalignant fibroblasts, formed tumors, which increased in tumor volume over time (Figure 1b–e). The engraftment of human cells into fish larvae was visualized by immunohistochemistry (IHC) staining on zebrafish sections injected with SK-N-BE(2)-C cells using STEM121 antibody, which reacts specifically with a human cytoplasmic protein (Figure 1f). 

In addition, we performed IHC staining against macrophage-specific protein Iba1 and primitive macrophage marker Csf1r to assess scale of immune cell infiltration (Figure S1b). Our results indicate that while macrophages are present at the implantation site, the vast majority of cells in the tumor mass are human tumor cells. IHC analysis of two independent markers specific for cells undergoing mitosis, Ki-67 and phosphorylated histone H3 (pHH3), revealed that 40–50% of engrafted SK-N-BE(2)-C cells, 60–70% of engrafted HD-N33 cells, and 40–50% of engrafted NB-S-124 cells were mitotically active following implantation (Figure 1g). Comparison with tissue slides obtained from human neuroblastoma patient samples indicated a similar proliferation rate of approximately 40% of tumor cells per slide (Ki-67 staining, Figure 1g). Moreover, single stack analysis and co-staining with TMRE (mitochondrial marker for living cells) of tumor cells injected into the yolk sac confirmed cellularity and the detection of living cells on experimental day 3 (Figure 1c,d). Overall, these data indicate that, following engraftment into the yolk sac of zebrafish embryos, pediatric tumor cells survive and proliferate at rates similar to those observed in patient tumors. 

### 2.2. Toxicity Studies and Determination of In Vivo Activity of HDAC Inhibitors

Before testing drug efficacy in zebrafish xenografts, optimal drug concentrations and maximal tolerated doses were determined. The applied drugs were broad-spectrum HDACis (panobinostat, vorinostat), which are already FDA-approved for T cell lymphoma and in clinical trials for childhood tumors [41], and one class-selective HDACi, tubastatin A, which is a selective class IIb inhibitor that sensitizes resistant neuroblastoma cells to doxorubicin treatment as shown by our previous work [29,31]. Toxicity tests, i.e., treatment of fish larvae for three days without injection of tumor cells, were performed to determine the maximal tolerated dose (MTD) and lethal dose (LD) for each compound used (Figure S2a, Table 2). The highest concentration that could be used without observable morbidity, changes in morphology, or severe aberrations of larval behavior was considered the MTD (Table 2). 

**Figure 2:**
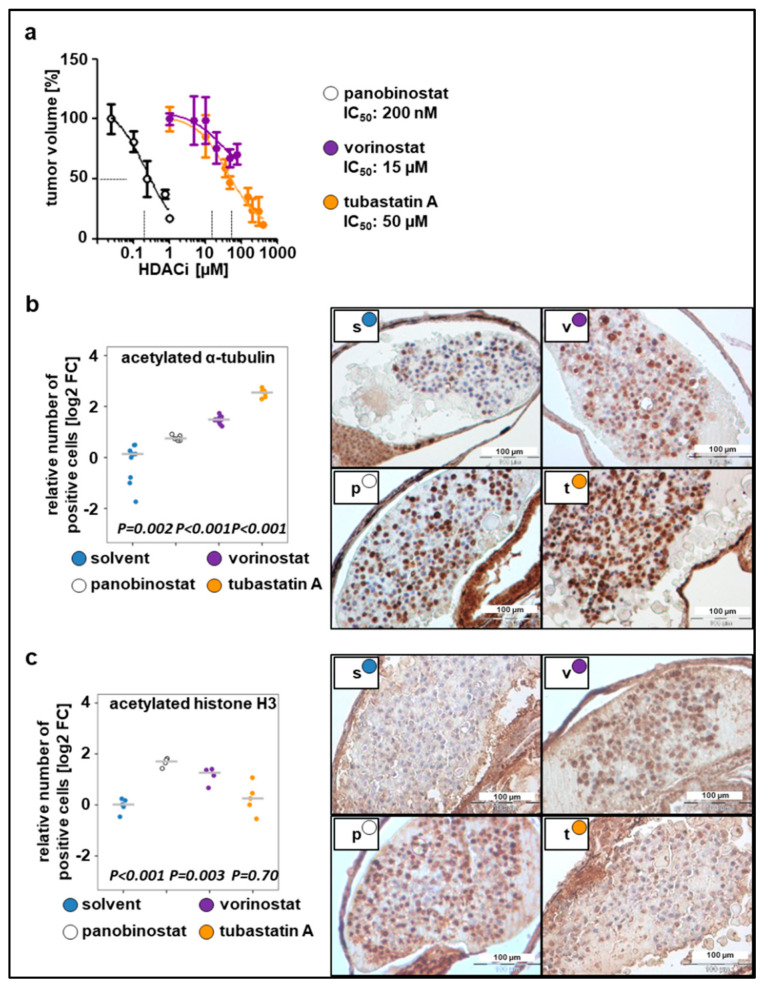
Dose responses to the tested compounds. (**a**) Relative IC_50_ values calculated based on the change in tumor volume following 48 h treatment with tested compounds, as compared to the solvent. *n* = 3–5 fish per concentration, values are mean ± SEM. (**b**) Left: quantification of staining for acetylated α-tubulin. Right: representative images of immunohistochemical staining (**c**) Left: quantification of staining for acetylated histone H3. Right: representative images of immunohistochemical staining. (**b**–**c**) The number of positive cells was calculated as the ratio of positive to total cells counted, relative to solvent and displayed as log2 fold change (FC) versus solvent in a strip chart. Median is displayed as a grey line. One-way ANOVA with Tukey’s multiple comparison test, scale bar = 100 µm. s, solvent; v, vorinostat (15 µM); p, panobinostat (200 nM); t, tubastatin A (50 µM).

To determine optimal drug concentrations, larvae bearing xenografted tumor cells were incubated with increasing drug concentrations 24 h post implantation (hpi), ranging from the concentrations used in previous cell culture experiments to the MTD_(larvae)_ (Figure 2a). Relative IC_50_ values, determined based on the change in tumor mass volume, were up to 20-fold higher than the concentrations used in cell culture studies since substances were applied to the surrounding water to be absorbed by the larvae. This observation is in line with the literature, which estimates that the extent of compound absorption by zebrafish larvae is 1/10 to 1/20 of the cell culture treatment concentration [13,42]. The determined IC_50_ values were as follows: 50 µM for tubastatin A, 200 nM for panobinostat, and 15 µM for vorinostat (Figure 2a). Using these concentrations, we evaluated whether HDACis were able to reach their specific enzymatic targets. Exposure of larvae bearing SK-N-BE(2)-C xenografts to broad-spectrum HDACis for 48 h increased the number of cells that stained positive for ac-tubulin, the substrate of HDAC6, and ac-histone H3, the substrate of HDAC1–3. Treatment with tubastatin A, a selective inhibitor of HDAC6/10, resulted in a 5-fold increase of acetylated α-tubulin but not acetylated H3, indicating on-target activity on HDAC6/10 but not off-target activity on HDAC1–3 at the applied concentration of 50 µM (Figure 2b,c). Together, these results suggest that larvae absorb sufficient HDACi for its intended on-target effects to occur in zebrafish neuroblastoma xenografts.

### 2.3. Zebrafish Xenograft Model Identifies Treatment Combinations Involving Doxorubicin and Selected HDAC Inhibitors as Promising Strategies for Neuroblastoma Therapy

**Figure 3:**
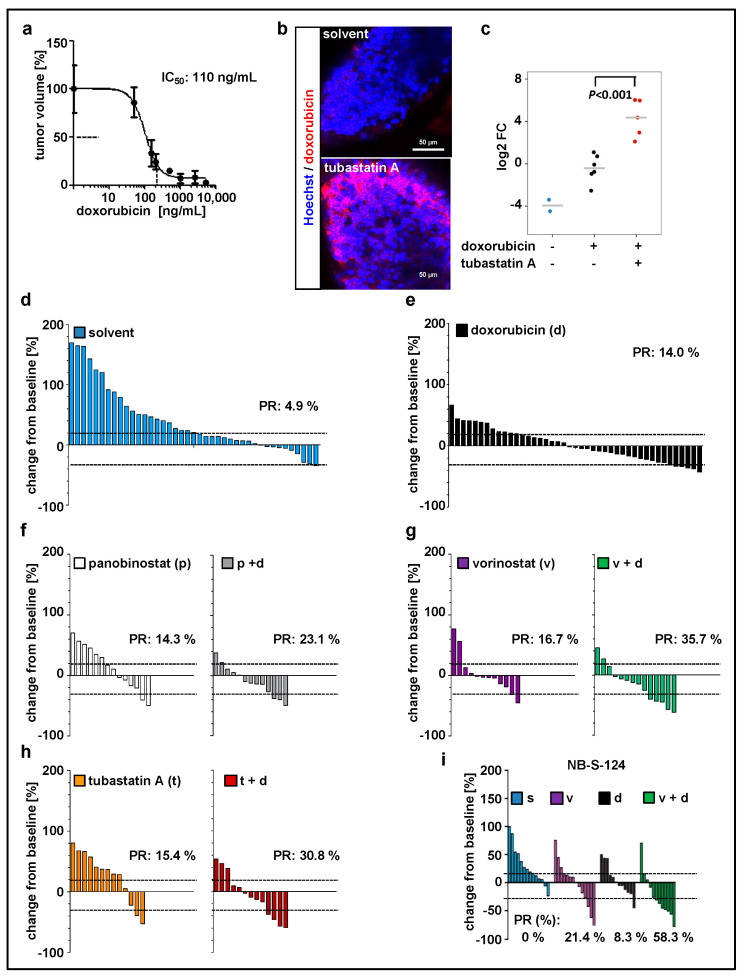
Zebrafish xenograft model identifies treatment combinations involving doxorubicin and selected histone deacetylase (HDAC) inhibitors as promising strategies for neuroblastoma therapy. (**a**) Relative IC_50_ value calculated based on the change in tumor volume following 48 h treatment with doxorubicin, as compared to the solvent. *n* = 3–5 fish per concentration, values are mean ± SEM. (**b**,**c**) Representative images (**b**) and quantification (**c**) of zebrafish larvae engrafted with Hoechst stained and doxorubicin-treated SK-N-BE(2)-C cells. Larvae, except of unstained larvae, were exposed for 48 h to doxorubicin (red fluorescence) and tubastatin A or solvent. Red fluorescence of doxorubicin was visualized by confocal microscopy and normalized to tumor area. Results are presented as log2 fold change (FC) relative to doxorubicin alone. Strip charts, median is displayed as a grey line. One-way ANOVA with Tukey’s multiple comparison test, scale bar = 50 µm. (**d**–**h**) Waterfall plots demonstrating change in tumor volume (%) for each individual xenograft, from baseline (day 1 = start of the treatment) to day 3 yolk sac after implantation of SK-N-BE(2)-C tumor cells. Dotted lines are drawn according to Response Evaluation Criteria in Solid Tumors (RECIST) 1.1 adopted for zebrafish tumors, to visualize best response: progressive disease, at least a 20% increase in tumor volume; partial response (PR), at least a 30% decrease in tumor volume; *n* = 12–17 larvae per group from at least two independent experiments; each bar reflects one individual xenograft. (**i**) Waterfall plots demonstrating change in tumor volume (%) for individual zebrafish larvae engrafted with NB-S-124 cells, from baseline (day 1 = start of the treatment) to day 3 after tumor implantation. Numbers indicates the percentage of larvae with partial response (PR) in each treatment group on day 3. *n* = 12–14 zebrafish per group. (**b**–**i**) Concentrations used: doxorubicin 110 ng/mL, vorinostat 15 µM, panobinostat 200 nM, tubastatin A 50 µM.

Since doxorubicin is one of the most commonly used chemotherapeutics in neuroblastoma treatment [43], we used our model to evaluate the treatment with doxorubicin in combination with HDACi. Before that, the maximal tolerated dose (Figure S2b, Table 2) and optimal drug concentration (Figure 3a) were determined. We have previously demonstrated that inhibition of HDAC6/10 using tubastatin A results in intracellular accumulation of doxorubicin and thus enhances neuroblastoma cell death upon combination treatment [31]. Likewise, in our xenograft model, tubastatin A increased doxorubicin accumulation in zebrafish tissues and engrafted tumor cells (Figure 3b,c). Additionally, these results demonstrate absorption of doxorubicin from the surrounding buffer solution by zebrafish larvae and confirm the delivery of doxorubicin to the implanted cells. Doxorubicin has an intrinsic fluorescence due to the presence of a hydroxy-substituted anthraquinone chromophore. For our SK-N-BE(2)-C model, we then tested panobinostat, vorinostat, and tubastatin A alone and in combination with doxorubicin (Figure 3d–h; Figure S3a). Data were expressed as waterfall plots comparing change in tumor volume over baseline of individual fish larvae. The treatment with doxorubicin in combination with HDACi substantially improved the response rate (PR = partial response according to RECIST) with a 23% PR for panobinostat and doxorubicin, 31% for tubastatin A and doxorubicin, and 36% for vorinostat and doxorubicin (Figure 3). We successfully validated the treatment combination consisting of vorinostat plus doxorubicin with the short-term cultured primary NB-S-124 cells derived from a neuroblastoma patient. In this experiment, nearly 60% of individual xenografts reached PR (Figure 3i; Figure S3b). Of note, MYCN nonamplified cells, such as GI-MEN neuroblastoma cells, do not seem to be as responsive to this treatment as the two MYCN-amplified models (Figure S3d). In summary, our zebrafish model can be used to identify effective, novel in vivo treatment combinations.

### 2.4. Combination Therapy with Vorinostat Activates Caspase-3 in Engrafted Tumors 

**Figure 4:**
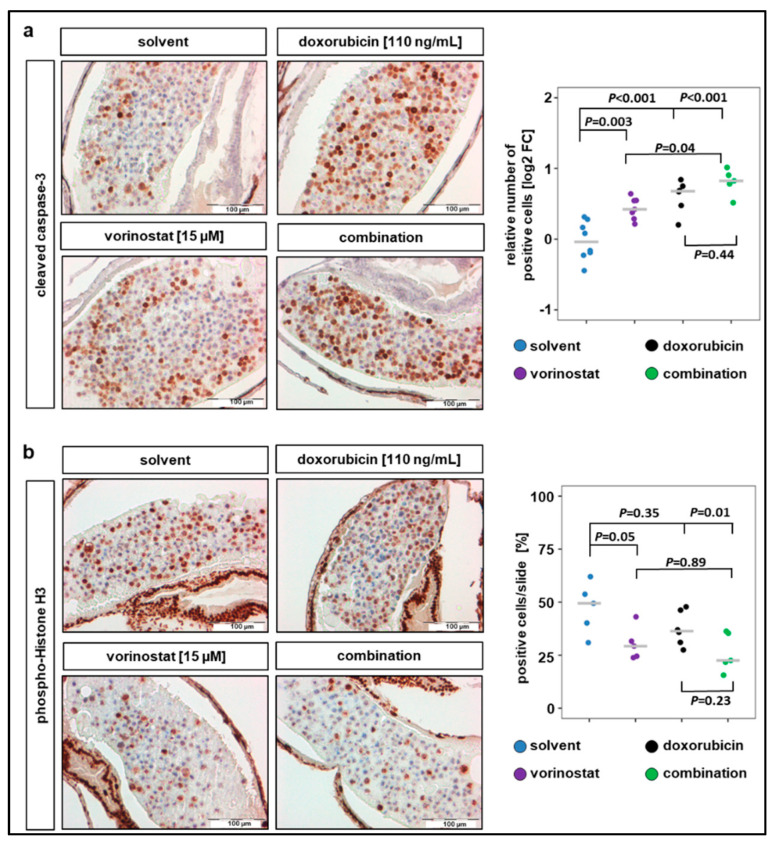
Combination therapy of doxorubicin with vorinostat increases caspase-3 cleavage and reduces number of mitotic tumor cells. (**a**) Treatments for 48 h with vorinostat, doxorubicin, or combination of both increased the amount of cleaved caspase-3 positive SK-N-BE(2)-C derived tumor cells. Left panel: representative images of sections stained against cleaved caspase-3. Right panel: results are presented as log2 fold change (FC) in a strip chart, median is displayed as a grey line. The number of positive cells was calculated as the ratio of positive to total cells counted, relative to solvent. One-way ANOVA with Tukey’s multiple comparison test, solvent vs. vorinostat, scale bar = 100 µm. (**b**) Left panel: representative images of sections stained against phospho-histone H3, a marker specific for cells undergoing mitosis. Right panel: strip chart, median is displayed as a grey line. The number of positive cells (%) was calculated as the ratio of positive to total cells counted. One-way ANOVA with Tukey’s multiple comparison test, scale bar = 100 µm.

IHC analysis of the xenograft sections revealed that a 48 h exposure of SK-N-BE(2)-C zebrafish xenografts to vorinostat, doxorubicin, or a combination of both increased the number of cells positive for cleaved caspase-3 (Figure 4a). All treatment conditions significantly increased the number of cells with cleaved caspase-3 compared to solvent-treated control, and the highest increase was observed in cells treated with combination therapy (Figure 4a). In contrast, the number of cells positive for the proliferation marker pHH3 only significantly decreased when combination treatment was applied (Figure 4b). The average number of tumor cells per slide positive for pHH3 was 47% for solvent-treated, 30% for vorinostat alone, 38% for doxorubicin alone, and 26% for vorinostat and doxorubicin combined. Due to this relatively short treatment duration (48 h), the apoptosis induction of the combination therapy is predominant over the antiproliferative effect. Together, these results demonstrate that treatment with HDACi–doxorubicin combinations promotes tumor regression, possibly by inducing proapoptotic pathways.

### 2.5. Tumor Cell Dissemination in the Zebrafish Xenograft Model of Neuroblastoma 

**Figure 5:**
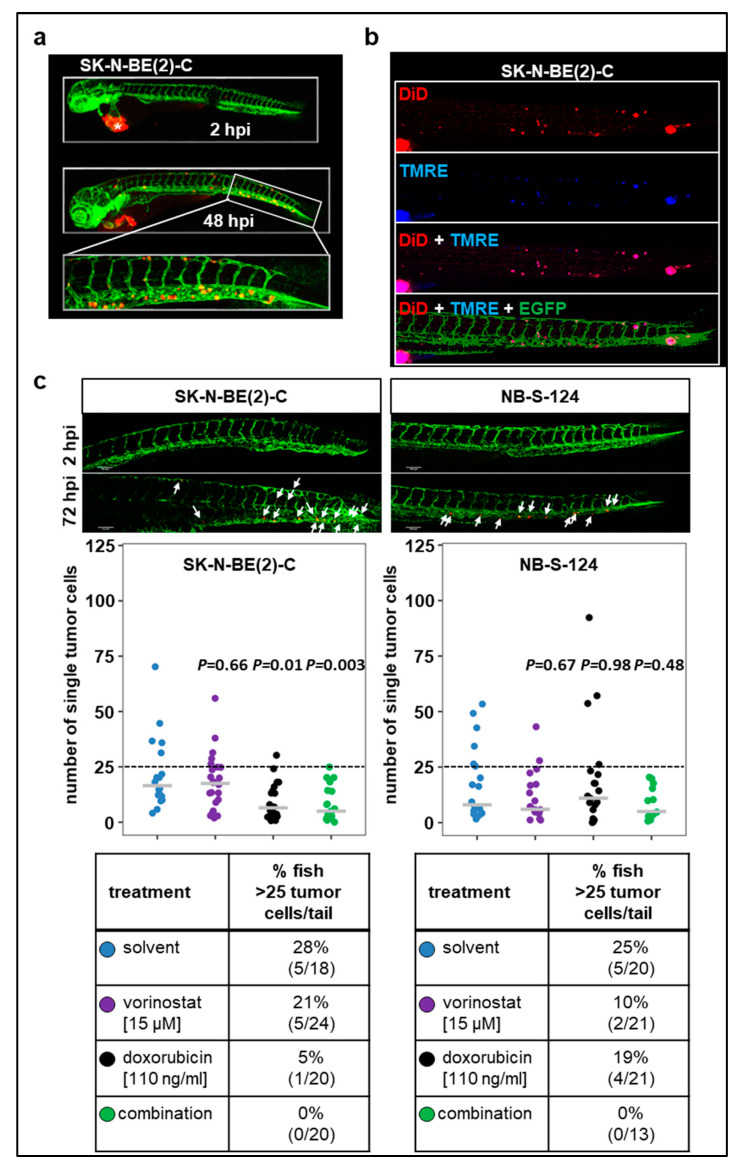
Tumor cell dissemination zebrafish xenograft models (**a**) Representative images including the tail region of SK-N-BE(2)-C cells at 2 and 48 hpi. * primary tumor. (**b**) TMRE dye is sequestered by active mitochondria, which is indicative of living cells. Representative images of zebrafish larvae (72 hpi) injected into perivitelline space with a suspension of SK-N-BE(2)-C cells labeled with TMRE (blue) and DiD (red). (**c**) Representative images of tail disseminated cells of SK-N-BE(2)-C or NB-S-124 cells at 2 and 72 hpi. Scale bar = 100 µm. Strip charts displaying absolute numbers of single tail-disseminated SK-N-BE(2)-C and NB-S-124 cells at 72 hpi. White arrows: tumor cells. Tables represent absolute numbers and percentages of fish with more than 25 tail-disseminated cells per treatment. One-way ANOVA with Tukey’s multiple comparison test.

Metastasis is the major cause of death in cancer, including neuroblastoma. Therefore, we further characterized our zebrafish xenograft models in terms of potential dissemination by injecting CM-DiI-labeled SK-N-BE(2)-C and NB-S-124 cells into the perivitelline space (Figure 5). In both models, numerous disseminating tumor cells in the tail region were revealed at 72 hpi. 

Additionally, we injected tumor cells that were double-stained with TMRE and DiD into the perivitelline space (Figure 5b). The majority of cells detected in the tail region displayed both TMRE and DID fluorescence. Together, these experiments indicate that viable human tumor cells are present in the tail region of zebrafish larvae following the engraftment into perivitelline space. Absolute numbers are plotted in Figure 5c. To further assess the ability of the cells to disseminate and metastasize, we set a threshold of more than 25 tail-disseminated single cells. In SK-N-BE(2)-C xenografts, single treatment with doxorubicin substantially reduced the number of fish with more than 25 tail-disseminated single cells, which was further reduced by the combination therapy. Results obtained with SK-N-BE(2)-C were confirmed in short-term culture of NB-S-124 cells (Figure 5c). 

In conclusion, the xenograft models described here allow for effective visualization and quantification of disseminating tumor cells. The present study demonstrated that combination therapy involving vorinostat and doxorubicin substantially attenuated progression of primary tumors in zebrafish larvae and showed decreased tumor cell dissemination in both neuroblastoma models. 

## 3. Discussion

The unique value of zebrafish as a model in cancer research for developing new therapies is based on their high-throughput capacity combined with whole-organism biology [44,45]. Although certain shortcomings have to be accepted, zebrafish as a disease model is a powerful tool overall and may lead to great advancement in precision oncology and personalized medicine [44,46]. Phenotype-based drug discovery in zebrafish through screening of large compound libraries often allows for identification of unanticipated drug candidates [45]. In fact, nearly ten compounds that were identified through zebrafish screens have already entered the clinic [44]. Here, we present, in addition to the recently published neuroblastoma brain tumor model [47], for the first time yolk sac and perivitelline space migration zebrafish xenograft models of neuroblastoma. Moreover, we demonstrate their value in early drug testing with the ultimate goal of preselecting the most promising therapies including drug combinations. The ability to track cancer formation and progression in real time with single-cell resolution in an intact microenvironment makes zebrafish an excellent xenograft model. We demonstrate that engrafted tumor cells maintained sufficient proliferation rates, allowing evaluation of efficacy of selected drugs within a short time frame. 

One continuing challenge when establishing zebrafish xenograft models concerns the temperature in which fish should be kept after implantation of human material. While the physiological temperature for zebrafish is 28 °C, it is increased to 32.5–35 °C after transplantation of human cells in most published studies [18,21,42,48,49]. Based on literature reports indicating that human cancer cells are able to proliferate in temperatures below the optimal 37 °C [42,48,49], zebrafish xenografts were kept at 34 °C in the present study. This temperature did not induce observable developmental abnormalities or toxicity in fish; at the same time, it allowed sufficient tumor cell maintenance. However, it could be argued that the behavior of transplanted tumor cells was altered under these conditions. Recently, it has been reported that human cancer cells in zebrafish xenografts can be maintained at 36 °C or even 37 °C [12,50]. The authors report that this temperature improved proliferation rates of transplanted cells, providing more suitable conditions for drug testing without causing noticeable toxicity. While we have performed several experiments at 36 °C, this temperature was associated with dramatically increased toxicity in our model, reaching a mortality as high as 67% of fish larvae. It is possible that successful incubation of the xenografts at 36 °C could be achieved following further optimization of the experimental procedure, e.g., gradual acclimatization to an ambient temperature of 36 °C. Such approaches should be considered for future studies.

One of the main advantages of zebrafish models is that they allow for drug efficacy testing in an intact organism with a liver, kidneys, and tissue barriers that are functioning from early development, in addition to physiological drug absorption, metabolism, and excretion [51]. However, since the compounds are added into ambient water in which larvae are swimming, amount of drug exposure is difficult to control [11,12,52]. To address these concerns, first we measured relative IC_50_ values for the drugs used in the present study. The concentrations determined as IC_50_ ranged from 1.5- to 20-fold higher than the concentrations used in cell culture studies, which have been previously published [13]. Secondly, we verified whether HDACis were reaching their enzymatic targets by measuring the expression of acetylated α-tubulin and acetylated histone H3. For example, our analysis confirmed specific on-target activity for tubastatin A, an inhibitor targeting the IIb class of HDACs. This class of HDAC is enriched in the cytoplasm and primarily targets nonhistone proteins [53], and there was no observed off-target impact of tubastatin A on histones. Nevertheless, it remains difficult to directly compare the concentrations used with our model with clinically achievable concentrations. This could be optimized in future studies by determination of intratumoral drug concentrations through MALDI-MS analysis, after sacrificing zebrafish larvae at different time points (2, 6, 24 and 48 h).

To further test our model as a tool for preselecting promising therapies, we evaluated the efficacy of doxorubicin, one of the most commonly used chemotherapeutics in neuroblastoma treatment, alone and in combination with broad-spectrum HDACis in clinical use or class IIb selective HDACis. We found that therapies involving doxorubicin in combination with panobinostat, vorinostat, or tubastatin A significantly reduced tumor volumes. Indeed, we and others have reported previously that pharmacological inhibition of HDACs can enhance the efficacy of anticancer agents, including topoisomerase II inhibitors, through induction of DNA damage and activation of intrinsic apoptotic pathways in tumor cells [29,31,54,55]. A growing body of preclinical and early clinical evidence supports vorinostat, which is already approved for cutaneous T-cell lymphoma therapy, as a promising agent for various combination treatments for neuroblastoma [56,57,58]. Further analysis of zebrafish xenografts revealed increased cleaved caspase-3, suggesting activation of caspase-dependent programmed cell death in tumor cells following exposure to the combination therapy with vorinostat and doxorubicin. As other types of cell death might also exist, additional read-outs would be necessary to further confirm apoptosis as the predominant type of cell death occurring. Admittedly, subsequent mechanistic studies are challenging, since the tumor consists of only a few cells (150–200 cells), which cannot easily be isolated and characterized through Western blotting or gene expression analysis. 

Additionally, we confirmed for the first time in vivo that selective inhibition of HDAC6/10 induces intracellular doxorubicin accumulation in zebrafish tissues and engrafted tumor cells, an effect we had observed previously only in cell culture [31]. The combination of vorinostat and doxorubicin effectively attenuated growth of primary neuroblastoma tumors and was also able to affect the dissemination potential. At the same time, we demonstrated that our zebrafish xenograft model of neuroblastoma allows tracking of injected tumor cells and quantification of tumor cell dissemination, making it a relevant model for evaluating the antimetastatic potential of various therapeutic strategies. 

In summary, our work describes a rapid preclinical zebrafish xenograft model of neuroblastoma that predicts standard drug testing outcomes within one week, which is much faster than the weeks it takes for PDX mouse models to grow. Within this short time frame, it would be in principle possible to generate PDX results for a single patient, which could be discussed by the oncologists and therefore might affect the treatment options for the patient. Introduction of innovative automation technologies, e.g., embryo sorting, microinjections, and drug dispensing, could further advance efficiency and high-throughput potential of this model. 

## 4. Materials and Methods 

### 4.1. Cell Lines and Culture Conditions

Human neuroblastoma cell line SK-N-BE(2)-C was obtained from the European Collection of Authenticated Cell Cultures (ECACC, London, UK). The human neuroblastoma cell line GI-MEN was obtained from DSMZ German Collection of Microorganisms and Cell Cultures GmbH, Germany. GI-MEN cells are MYCN nonamplified, TP53 wild-type, but have a 1p deletion. The cells were cultured in RPMI medium (Gibco/ThermoFisher, Braunschweig, Germany, REF 21875-034) containing L-glutamine, supplemented with 10% fetal calf serum (FCS, Sigma-Aldrich, St. Louis, MO, USA). The cells were mycoplasma negative (routinely checked with PCR every month) and were regularly checked for a broad range of contaminations by Multiplexion, Heidelberg, Germany. Both cell lines were verified using DNA fingerprinting authentication by the DSMZ, Braunschweig, Germany.

### 4.2. Patient-Derived Primary Cells

Primary neuroblastoma cells HD-N33 and NB-S-124 (NB8) cells were described previously [37]. Briefly, the cells of the patient sample were briefly propagated and kept as a spheroid culture in serum-free stem-cell medium (Neurobasal) supplied with growth factors EGF and FGF. Vials of this original culture were frozen down. The thawed cells could be used for a short time period in culture. NB-S-124 cells were maintained in Neurobasal-A Medium (Gibco/ThermoFisher) supplemented with L-glutamine (Sigma-Aldrich) and penicillin/streptomycin (Sigma-Aldrich), as well as epidermal growth factor (EGF, 20 ng/mL) (PromoCell, Heidelberg, Germany), fibroblast growth factor 2 (FGF2, 20 ng/mL)(Peprotech, Rocky Hell, New Jersey, USA), heparin sodium salt (2 µg/L) (Sigma-Aldrich), and B27 (Gibco/ThermoFisher). HD-N33 cells were cultured in Roswell Park Memorial Institute medium (RPMI, Gibco/ThermoFisher) containing L-glutamine (Sigma-Aldrich) and supplemented with 10% fetal FCS (Sigma-Aldrich, Munich, Germany) and 1% NEAA (Lonza Group, Basel, Switzerland). All cells were mycoplasma negative and were checked for a broad range of contaminations by Multiplexion, Heidelberg, Germany.

### 4.3. Patient Material

Paraffin-embedded sections of human neuroblastoma tumors (project number: 2500) were provided by the tissue bank of the National Center for Tumor Diseases (NCT, Heidelberg, Germany) in accordance with the regulations of the tissue bank and the approval of the ethics committee of Heidelberg University. 

### 4.4. Ethical Approval 

Experiments involving human patient material were performed in accordance with the Declaration of Helsinki and were approved by the ethics committee of the Medical Faculty University Hospital Heidelberg. All applicable national and institutional guidelines for the care and use of zebrafish were followed. All procedures performed involving animals were in accordance with ethical standards of the institution.

### 4.5. Zebrafish Lines

Zebrafish husbandry (permit number 35-9185.64/BH Hassel/Meder) and experiments (permit number 35-9185.81/G-126/15) were performed according to local animal welfare standards (Tierschutzgesetz §11, Abs. 1, No. 1) and in accordance with European Union animal welfare guidelines (EU Directive 2010/63/EU). Adult zebrafish were maintained on a 14-h light and 10-h dark cycle at 28.5 °C in a recirculation system (Schwarz Ltd., Germany; Mueller and Pfleger Ltd., Germany). Mature fish were fed daily with a combination of freshwater aquarium flake food (TetraWerke, Melle, Germany) and live artemia shrimps (Sanders Great Salt Lake Artemia Cysts). The TE4/6 wild-type strain [59] (tumor volume experiments) or the Tg (fli1:EGFP) line [60] (dissemination experiments), which uniformly expresses EGFP throughout their vasculature, were used for breeding. Briefly, sexually mature zebrafish were kept overnight in mating tanks (pairwise), separated by a partition plate. The partition was removed in the beginning of the light phase and spawning began shortly after. Eggs were collected, rinsed, and placed in a petri dish with 1X E3 embryonic buffer. Embryos were raised in an incubator at 28.5 °C. At 24 h post fertilization (hpf), the buffer was exchanged with 1X E3 embryonic buffer supplemented with 0.2 mM PTU (Sigma). 

### 4.6. Cell Preparation and Zebrafish Xenotransplantation 

Adherent cells SK-N-BE(2)-C and HD-N33 were cultured to 70–80% confluence, then washed once with PBS (Lonza, Basel, Switzerland) and dissociated using trypsin (Gibco) or versene (pH 8.0, EDTA disodium salt purchased from GERBU Biotechnik GmbH, Heidelberg, Germany), respectively. NB-S-124 cells, which grow as spheroids, were dissociated using versene and by pipetting up and down several times until a homogeneous, single-cell suspension was obtained. Cell counts, as well as cell viability, were measured by automated trypan blue staining with Vi-Cell XR Cell Viability Analyzer (Beckman Coulter, Krefeld, Germany). Briefly, Vi-Cell XR utilizes video capture technology and sample handling, and the mean number of viable cells per sample was determined based on the analysis of 50 captured images. After counting, cells were centrifuged (230× *g*, 5 min), resuspended in phenol red free RPMI medium, and labeled with CellTracker CM-DiI (Thermo Fisher Scientific, Waltham, MA, USA) for 5 min at 37 °C and then 15 min at 4 °C. CM-DiI is a cell-permeable dye that is transferred to daughter cells but not adjacent cells in a population and therefore is well suited to track engrafted material. Co-staining with Vybrant DiD Cell-Labeling Solution (Thermo Fisher Scientific) and tetramethylrhodamine ethyl ester (TMRE) (Thermo Fisher Scientific) was performed by incubating cell suspension for 10 min at 37 °C and then 20 min at 4 °C. TMRE is a cell-permeable fluorescent dye that is readily sequestered by active mitochondria and serves as a marker for viable cells, while DiD is an analog of CM-DiI with markedly red-shifted fluorescence excitation and emission spectra. To minimize cell clumping, DNase I (18 Kunitz units/mL, Sigma) was added to the cell suspension. Following the incubation, SK-N-BE(2)-C and HD-N33 cells were washed twice with 10% FCS RPMI and twice with serum-free RPMI, while NB-S-124 cells were washed four times using serum-free RPMI. Finally, cells were resuspended in serum-free RPMI to a final concentration of 1.0 × 10^8^ cell/mL. 

Before implantation, embryos were dechorionized if necessary, anesthetized with tricaine (0.02%, Sigma), and embedded in lateral position in 1.0% low-gelling-temperature agarose (Sigma). A total of 150–250 CM-DiI-labeled tumor cells were injected into the yolk sac (for tumor volume studies) of each zebrafish larva at 48 h post fertilization (hpf) using FemtoJet express microinjector (Eppendorf, Hamburg, Germany) and glass microinjection needles (Science Products, Hofheim, Germany). For the analyses of cell dissemination, approximately 100 CM-DiI-labeled tumor cells were injected into the perivitelline space. Injected larvae were washed with E3 buffer, transferred to plates containing E3 buffer supplemented with PTU, and placed at 28.5 °C. One hour after tumor cell injection, larvae were transferred to 34 °C. The xenografts were evaluated by fluorescence microscopy (Olympus, Tokyo, Japan) 2 h post implantation (hpi). Only larvae with red fluorescence at the injection site were used for further experiments.

Of note, our tumor cells were maintained at 37 °C before injection without step-wise adaptation to 34 °C, which is ambient temperature for fish, in contrast to many zebrafish xenograft protocols.

### 4.7. Confocal Analysis of Tumor Cell Proliferation and Treatment Efficacy in Zebrafish Xenografts

To monitor proliferation of tumor cells in the zebrafish host, injected larvae were imaged using confocal microscopy on the day that injections were performed (day 0), then daily thereafter for three days (days 1, 2, and 3). Images of living larvae were obtained using Zeiss LSM 710 confocal microscope (Zeiss, Oberkochen, Germany) equipped with the 10×/0.3 EC Plan-Neofluar and 20×/0.8 Plan-Apochromat objectives, Argon 514 nm laser line for DiI, standard PMT for fluorescence detection and T-PMT for transmitted light controlled by ZEN software (Zeiss). The microscope settings were identical for each image acquisition. For imaging, larvae were anesthetized with tricaine (0.02%, Sigma) and embedded in lateral position in 1.0% low-gelling-temperature agarose (Sigma) in chambered coverslips (ibidi, Martinsried, Germany). At the end of the experiments, zebrafish larvae were euthanized with 1% sodium hypochlorite at the zebrafish facility in the medical facility of University of Heidelberg. To determine tumor progression, we developed in-house a macro for FIJI software (a version of ImageJ) [61] and used it to quantify tumor volume. Images were analyzed automatically and all settings were identical for each xenograft. The macro duplicated the channel of interest from the 3D image hyperstacks and analyzed volumes of objects in the user-defined area using ImageJ’s 3D Object Counter tool. It also displayed results using 3D Project tool for the visual inspection at the end. All the parameters in the 3D Object Counter were kept constant during the analysis. The 3D image hyperstacks were used to determine tumor volumes, and results were displayed for manual inspection at the end. 

To evaluate treatment efficacy, tumor growth was monitored by confocal microscopy before the drug exposure and 48 h post treatment. The images were analyzed automatically using a macro developed for FIJI software as described above. Relative IC_50_ values were calculated using GraphPad Software version 5.0 (La Jolla, CA, USA) based on the change in tumor volume following treatment with tested compounds and compared to the solvent-treated control. In further experiments, 24 hpi selected xenografts were exposed to freshly prepared E3 medium (supplemented with PTU) containing drugs at determined IC_50_ concentrations or to solvent. Confocal images of tumor growth were obtained and assessed as described above. Response of tumor volumes were categorized according to Response Evaluation Criteria in Solid Tumors (RECIST) 1.1, adopted for zebrafish tumors, to visualize best response: progressive disease (PD, at least a 20% increase in tumor volume), stable disease (SD, neither sufficient shrinkage to qualify for PR nor sufficient increase to qualify for PD), and partial response (PR, at least a 30% decrease in tumor volume).

### 4.8. Confocal Analysis of Tumor Cell Dissemination and Treatment Efficacy in Zebrafish Xenografts

A suspension of CM-DiI-labeled tumor cells or a suspension of tumor cells co-stained with DiD and TMRE was injected into the perivitelline space of larvae 48 hpf. Zebrafish larvae were imaged immediately following injection, as well as at 72 hpi, using confocal microscopy as described above. To visualize disseminating tumor cells, we focused on the tail region of engrafted fish. Xenografts with tumor cells present in the tail region directly after injection were excluded because of erroneous injection into blood vessels rather than the perivitelline space. Engrafted larvae were exposed to selected treatments at determined concentrations starting at 24 hpi with a total incubation time of 48 h. To automate analysis and exclude personal bias, we developed a FIJI macro for counting the absolute number of disseminating single tumor cells. Briefly, the macro created maximum Z-projection of 3D stacks, filtered images using “Despeckle” tool, and segmented and counted cells above intensity threshold using “Find Maxima and Analyze Particles” tool with the minimum object size set to 20 µm^2^. All the image acquisition and processing parameters were kept constant during the analysis.

### 4.9. Reagents for Tumor Treatment

Tubastatin A (Biozol, Eching, Germany; 10 mM stock), vorinostat (Selleck Chemicals, Munich, Germany; 1 M stock), and panobinostat (Cayman Chemical, Hamburg, Germany; 50 mM stock) were dissolved in DMSO. Doxorubicin (Merck Calbiochem, Darmstadt, Germany; 5.8 mg/mL stock) was dissolved in water. 

For treatment, larvae were incubated in freshly prepared buffer solution supplemented with 0.2 mM PTU, containing the drug of interest at increasing concentrations. Drug-containing solutions were administered at the beginning of the experiment and were not replaced during the incubation period. Solvent control was applied in the same manner as the drug treatments.

### 4.10. Immunohistochemistry (IHC)

Following fixation (24 h at 4 °C) in Special Fixative for Anatomy and Histology (Morphisto, Frankfurt am Main, Germany), xenografted zebrafish larvae were transferred to embedding cassettes equipped with biopsy pads, dehydrated, and infiltrated with paraffin. Briefly, fixed larvae were dehydrated using Leica ASP300S tissue processor (Leica Biosystems, Wetzlar, Germany) through incubation in aqueous solutions of increasing ethanol concentration (1 h in 50% ethanol, 2 h in 70% ethanol, 2 h in 80% ethanol, 1 h in 96% ethanol, 3 h in absolute ethanol) and thereafter incubated with xylene for 5 h. Next, samples were transferred to liquid paraffin at 60 °C and allowed to infiltrate for 5 h. Then, the larvae were embedded in paraffin, cut into 5-μm-thick sections, and mounted (five sections per slide) onto glass slides (Thermo Fisher Scientific). The sections were deparaffinized (xylene 2 × 5 min, ethanol absolute 2 × 3 min, 96% ethanol 1 × 3 min, 70% ethanol 1 × 3 min, 3 min in running cold tap water) and subjected to antigen retrieval at 100 °C in citrate buffer (pH 6.0). Endogenous peroxidase activity was suppressed by treatment with 3% H_2_O_2_ in Tris-buffered saline (TBS) (zebrafish xenografts) or 3% H_2_O_2_ in methanol (patient samples) for 20 min. The slides were washed with TBS, blocked with 3% bovine serum albumin (BSA, Sigma) in TBS, and incubated with primary antibody (diluted in 3% BSA in TBS) at 4 °C overnight, followed by incubation with secondary antibody (diluted in 3% BSA in TBS) for 1.5 h at room temperature. The following primary antibodies were used: Ki-67 (1:500; rabbit polyclonal, Abcam, Cambridge, UK), phospho-histone H3 (1:100, rabbit polyclonal, Zytomed Systems GmbH, Berlin, Germany), acetylated tubulin (1:100, mouse monoclonal, Sigma), acetylated histone H3 (1:200, rabbit polyclonal, Merck Millipore, Burlington, MA, USA), STEM121 (1:1000, mouse monoclonal, Takara Bio USA, Inc., Mountain View, CA, USA), cleaved caspase-3 (Asp175) (1:100, rabbit monoclonal, Cell Signaling Technology, Inc., Danvers, MA, USA), Iba1 (1:500, rabbit polyclonal, Wako Pure Chemical Industries, Osaka, Japan), and Csf1r (1:1000, rabbit polyclonal anti-zebrafish, Biozol Diagnostica). The following secondary antibodies were used: goat anti-mouse IgG-HRP (1:250, Jackson ImmunoResearch Laboratories, Inc., West Grove, PA, USA), goat anti-rabbit IgG-HRP (1:250, Sigma), and donkey anti-rabbit IgG-HRP (1:250, Invitrogen). DAB Substrate Kit (Abcam) was used for color developing. The sections were counterstained with hematoxylin and examined under a light microscope (Olympus). FIJI software and a macro program were employed for quantitative analysis of the xenograft slides, while the analysis of slides obtained from neuroblastoma patients was carried out manually. All quantitative analyses were performed in a blinded fashion on the xenograft slides (five tissue sections per zebrafish larva), which were obtained from at least two xenotransplantation replicates. 

### 4.11. Doxorubicin Accumulation in Engrafted Tumor Cells and Zebrafish Tissues 

SK-N-BE(2)-C cells were incubated with Hoechst nucleic acid dye (Thermo Fisher Scientific) at a concentration of 10 µg/mL for 30 min at 37 °C. Following the incubation, cells were washed three times with serum-free RPMI and were resuspended in serum-free RPMI to a final concentration of 1.0 × 10^8^ cell/mL.

Zebrafish larvae engrafted with Hoechst-stained SK-N-BE(2)-C cells were exposed to solvent, doxorubicin, or doxorubicin combined with tubastatin A for 48 h. Red fluorescence of doxorubicin was visualized by confocal microscopy. Doxorubicin has an intrinsic fluorescence due to the presence of a hydroxy-substituted anthraquinone chromophore, with typical excitation and emission maxima in the range of 500 nm and 552–585 nm, respectively. Total fluorescence intensity in the tumor area (determined by nuclear staining) was defined as that above the threshold set by the solvent group and was quantified using a macro program.

### 4.12. Statistical Analysis

GraphPad software in addition to R and R package CRAN ggplot2 [62,63] was used for statistical analysis. Data were analyzed using one-way analysis of variance (ANOVA) or one-way repeated measures ANOVA. For multiple comparisons, Tukey’s or Dunnett’s post hoc corrections were applied. Statistical probability of *p* < 0.05 was considered significant. The number of replicates for each experiment is depicted in the respective figure or figure legend. In all experiments, a single zebrafish larva was considered one replicate. No outliers were removed.

## 5. Conclusions

The zebrafish xenograft model of neuroblastomas allows for fast and reliable evaluation of toxicity and response of primary and metastatic tumors to treatments involving chemotherapy and novel drugs before testing in complex mammalian studies. 

## Figures and Tables

**Table 1:** Molecular characteristics of the tumor cell lines and patient-derived primary cells.

Name of the Cell Line/Patient-Derived Primary Cells	Cell Type	Molecular Characteristics
HD-N33	Primary neuroblastoma (short-term culture)	homozygous deletion of p16/p14, resemblance of mesenchymal subtype
NB-S-124 (NB8)	Primary neuroblastoma (short-term culture)	*MYCN* amplification and 1p deletion, wild-type *TP53* and p16/p14
SK-N-BE(2)-C	Neuroblastoma (established cell line)	*MYCN* amplification, *TP53* mutation

**Table 2:** Toxicity.

**Compound**	**Target**	**Concentration Range Tested**	**Maximum Tolerated Dose (MTD) ^1^**	**Lethal Dose (LTD) ^1^**
doxorubicin	chemotherapy	100–5000 ng/mL	500 ng/mL	1000 ng/mL ^2^
vorinostat	HDAC1–11	2.5–100 µM	100 µM	ND
panobinostat	HDAC1–11	0.1–20 µM	1 µM	10 µM ^3^
tubastatin A	HDAC6/10	12.5–200 µM	200 µM	ND

^1^ Three-day incubation time at 34 °C; ^2^ 1000 ng/mL: alive, but changed heart morphology; 5000 ng/mL: dead; ^3^ 10 µM: alive, but changed heart morphology; 20 µM: dead.

## Supplementary Materials

The following are available online at https://www.mdpi.com/1424-8247/13/11/345/s1, Figure S1: Neuroblastoma cell lines proliferate in zebrafish xenografts. (**a**) Scheme of experimental set up. Fluorescently labelled tumor cells were injected into the yolk sac of dechorionized larvae on experimental day 0 and from that time point on, the larvae were kept at 34 °C. Tumor cells were maintained at 37 °C before injection without step-wise adaptation to 34 °C. Brightfield picture: Day 0, tumor appears black. The treatment started on experimental day 1. Imaging of larvae was performed on experimental day 1 before treatment and on day 3 (the end of the experiment). (**b**) Representative image of zebrafish yolk sac engrafted with tumor cells (72 hpi), stained against macrophage specific protein Iba1. Zebrafish larvae implanted with SK-N-BE(2)-C cells (72 hpi) were stained against zebrafish macrophage and macrophage precursor specific cell surface receptor protein Csf1r. Scale bar = 50 µm. (**c**) Representative 10 × Tile region images of larvae, and 20 × and 40 × pictures of one focal plane of the z-stack acquired by confocal microscopy. CM-DiI labelled SK-N-BE(2)-C cells were injected into the yolk sac of zebrafish larvae 48 hpf. Imaging of larvae was performed 72 hpi. (**d**) TMRE is sequestered by active mitochondria which is indicative of living cells. Representative images of yolk sac of four different zebrafish larvae engrafted with SK-N-BE(2)-C cells co-stained with TMRE (blue) and DiD (red) (72 hpi). MIP: maximum intensity projection. Single stack: Pictures of one focal plane of the z-stack acquired by confocal microscopy. Scale bar: 100 µm. Figure S2: Toxicity test. Zebrafish larvae started receiving treatment on experimental day 1 (72 hpf) and larvae (*n* = 3 per concentration) were imaged using stereomicroscope on the day of treatment and following two consecutive days. Representative images of larvae treated with (**a**) HDACi (panobinostat, vorinostat and tubastatin A) and (**b**) doxorubicin. “Dead” states that the fish did not survive the treatment; “heart” implies an alteration in heart morphology. Figure S3: Zebrafish xenograft model identifies treatment combinations involving doxorubicin and selected HDAC inhibitors. Zebrafish larvae were imaged using confocal microscopy on day 3 after tumor cell implantation. Representative Tile region images of larvae injected with a suspension of fluorescently labeled SK-N-BE(2)-C cells (**a**) and NB-S-124 cells (**b**), tumor cells appear red. (**c**) Left: Waterfall plots demonstrating change in tumor volume [%] for individual zebrafish larvae engrafted with GI-MEN cells, from baseline (day 1 = start of the treatment) to day 3 after tumor implantation. Numbers indicate the percentage of larvae with partial response (PR) in each treatment group on day 3. *n* = 5–9 zebrafish per group. Right: Representative images of zebrafish yolk sac engrafted with CM-DiI labelled GI-MEN cells (72 hpi). The treatment started on experimental day 1 and larvae were imaged using confocal microscopy on experimental day 1 before treatment and on day 3. 20 × and 40 × images belong to the same larva in solvent treated group. Only one focal plane of the z-stack acquired by confocal microscopy is presented. Scale bar: 100 µm. Concentrations used: doxorubicin 110 ng/mL, vorinostat 15 µM.
